# A qualitative study of how Danish drug consumption rooms influence health and well-being among people who use drugs

**DOI:** 10.1186/s12954-016-0109-y

**Published:** 2016-06-16

**Authors:** Nanna Kappel, Eva Toth, Jette Tegner, Sigurd Lauridsen

**Affiliations:** Department of Nursing, Metropolitan University College, Copenhagen, Denmark; COWI, Kongens Lyngby, Denmark

## Abstract

**Background:**

Drug use contributes to higher rates of morbidity and mortality among people who use drugs compared to the general population. In 2012, Danish politicians passed a law that allowed drug consumption rooms (DCRs) to operate; among the objectives were to improve the well-being of vulnerable citizens and to reduce the number of overdoses. Five Danish DCRs are currently being operated. This article presents results from a national investigation focused on assessing the impact of Danish drug consumption rooms on the health and well-being of DCR clients and factors facilitating the acceptance of DCR clients in order to improve their health and refer them onward to social and health service providers.

**Methods:**

We conducted 250 h of participant observation in the DCRs, followed by in-depth qualitative interviews with 42 DCR clients and 25 staff members. Field notes and interviews were analysed and coded, and themes have been developed.

**Results:**

DCR clients experienced a sense of social acceptance while inside DCRs. Members of staff conveyed a welcoming, non-judgemental attitude, and DCR clients were predominantly satisfied with the facilities. They prioritized forging relations with drug users so as to foster a sense of social acceptance within DCRs. The primary goal of staff members is to prevent overdoses by informing clients about strong drugs and by intervening in cases of intoxication. DCRs provide security to clients. In cases of health-related problems, DCR clients were referred to local health clinics. Members of the staff build bridges for DCR clients by guiding them towards drug treatment programmes and services in the social and the health sectors.

**Conclusions:**

The study reveals a consistency between DCR clients and staff members with respect to appraisal of the importance of DCRs. Both clients and staff agreed that DCRs provide a safe haven in the environment in which DCR clients often live and that staff members’ approach to clients with the intention of promoting acceptance clears the path for the prevention and treatment of overdoses and providing referrals to healthcare facilities, to drug treatment centres and to social services.

## Background

To reduce harm and prevent overdoses (ODs) caused by unsafe drug use, a number of countries including Switzerland, Germany, Spain, Norway, Australia and Canada have established drug consumption rooms (DCRs) [1–3] over the last 20 years. Drug consumption rooms are defined as ‘professionally supervised healthcare facilities where drug users can use drugs in safer and more hygienic conditions’ [2]. A growing body of scientific evidence shows that DCRs have an impact on both improving health and reducing death by overdose among clients who use these facilities [4–13]. For people who use drugs, unsafe drug intake often involves unhygienic and incorrect injections which cause both injury and infection [14–18]. People who use drugs are overwhelmingly at risk of developing both acute and chronic illnesses, and they have a significantly higher rate of morbidity and mortality than the rest of the population [19].

Several qualitative studies highlight the benefits of DCRs [20]. DCRs address various contextual risks associated with public injecting enabling safer injection practices [21], providing refuge from street-based crime [22], mediating and facilitating access to healthcare and social resources [20] and delivering education regarding safer injection practices which is highly accepted among clients [6]. Thereby, DCRs offer numerous harm-reducing interventions [6]. Qualitative studies also show that the everyday contact between members of staff and clients and the intimacy that develops when clients inject in the presence of staff transforms the sensation of shame. It forges new connections and relationships and provides a social context in which the clients feel support [23]. DCR clients describe nursing staff of DCRs as non-judgemental. The staff address barriers among DCR clients to access care for injection-related injuries. Staff also facilitate access to healthcare by providing low-threshold nursing attention on site and connect with off-site medical attention [24].

The numbers of people who use drugs in Denmark dying from overdose and intoxication have increased over the last decade and harm-reducing policies such as opioid substitution treatment (OST) and needle exchange programmes have been implemented, but generally, politicians and health authorities have been against drug consumption rooms [25, 26]. In an act of civil disobedience, in 2011, Danish NGOs bought two old ambulances and opened the first mobile drug consumption rooms with nurses and doctors volunteering their professional services [27] until the law was changed. In 2012, Denmark followed the international lead and passed legislation that allow municipalities to establish DCRs. The motivation behind this political decision was threefold: to reduce the number of deaths by overdose, to improve life situations for people who use drugs by building bridges to the healthcare system, drug treatment facilities and social services, and to reduce the nuisance of public drug intake to surrounding neighbourhoods [28]. In the years following this landmark decision, three of the biggest cities in Denmark have established DCRs. Currently, more than 3564 people who use drugs are registered as clients of Danish drug consumption rooms [29].

The Danish drug consumption rooms follow two models of DCRs: either they are integrated units, typically part of a shelter with additional services such as counselling, laundry and shower facilities and a health clinic or mobile unit with limited space and only function as a hygienic, safe place for an injection. The Danish DCRs are financed by the municipalities and managed by NGOs except for the mobile DCR, which is directly financed and run by the Municipality of Copenhagen. The DCRs are run by the Men’s Home^1^ in Copenhagen and by the Danchurchsocial^2^ in both Odense and Aarhus. These are financed through provisional governmental funds, and at present, their future remains undecided. There are three DCRs in Copenhagen, one in Aarhus (second biggest city) and one in Odense (third biggest city). The drug scenes in the three different cities are different in regard to which drugs are prevalent locally. Around the drug scene at Vesterbro in Copenhagen, the preferred drug is cocaine which accounts for 60 % of reported drugs [29]. The DCR ‘Skyen’ in the Men’s Home has both a smoking section and a section for injection. This DCR is the busiest with a heterogeneity of the clients with many different nationalities. The Men’s Home also runs the other DCR located at Halmtorvet. This DCR has spaces for injection and has a health clinic in the same building. The mobile unit ‘Fixelancen’ is the smallest facility; it is parked close to Halmtorvet with places for injection.

The DCR in Odense is integrated in a shelter with place for injection. The clients in Odense are more homogeneous, and a lot of the clients have stable housing. The DCR in Odense is located near drug treatment services. In Odense, 39 % of the DCR clients prefer cocaine and 53 % prefer heroine [29].

The DCR in Aarhus is part of the shelter, and it has a section for injection and a small section for smoking. The clients in Aarhus are mainly people with many years of drug use. Ritalin is used by 40 % of the clients and next heroine accounting for 46 % of the drugs reported and only 1 % use cocaine [29].

All DCRs are staffed with health professionals, i.e. registered nurses (RNs) or nursing aides, who work together with social workers and social educators. All staff members have advanced first aid training and are trained in the effects and side effects of the most commonly consumed drugs. No additional formal training is required for employment at DCRs. The healthcare professionals are mainly responsible for intervention and treatment in cases of severe intoxication (see Table 1).

**Table 1:** Background information on the DCRs

Existing DCRs	Type of DCR	Opening hours	Opening days	Places injecting	Places smoking	Registered users (unique)	Users daily	Staff background
Aarhus	Integrated model: health clinic, shelter	7 h (8:00 am–3:00 pm)	Weekdays plus Saturdays	5	2	176	10–20	Nurses, nursing aides, social educator
Odense	Integrated model: health clinic, shelter	Five and a half hours (8:30 am–2:00 pm)	Weekdays plus Saturdays	5	0	296	30–40	Nursing aides, social workers, social educators
Copenhagen Skyen (The Cloud)	Integrated model: health clinic, shelter	23 h (6:00 am–5:00 am)	Every day	8	6	3092	300	Nurses, social educators
Copenhagen Halmtorvet	Specialized model: health clinic	6 h (11:30 am–5:30 pm)	Weekdays plus Saturdays	8	0		100	Nurses, social educators
Copenhagen Mobile DCR	Mobile	5 h (12:30 pm–5:30 pm)	Weekdays plus Saturdays	4	0	No registration	50	Nurses

To gain entry to DCRs, the clients must register and formally agree (by signature) to abide by the house rules (minors and pregnant women are prohibited from entry, no trade is permitted inside the facility and no client is allowed to provide assistance to peers). The DCRs vary as to how strictly the house rules are enforced; a temporary ban is the most severe sanction but is seldom used. During the registration process, the clients must accept that staff will intervene in case of overdose. Upon a client’s initial registration, he or she creates an alias, and at each entry into the DCR, the client indicates the type of drug that he or she plans to consume.

So far, Danish DCRs have been investigated in a smaller quantitative study [7], so this study was initiated to explore how Danish DCRs influence health and well-being among drug clients in the perspectives of both DCR clients and health professionals. More specifically, we investigate how the DCRs influence the well-being of the drug clients, the health and health-related behaviour of drug clients, and the access to, and use of, drug rehabilitation and primary and secondary healthcare facilities.

## Methods

### Data collection

This article covers the qualitative part of a mixed methods study that combined participant observation and semi-structured interviews with a quantitative questionnaire. In this study, we used a qualitative exploratory design drawing on ethnographic research methods in order to understand and interpret the detailed interactions between the staff and DCR clients. The researchers had informal talks with different professionals working in collaboration with the staff at the DCRs and explored the broader service context in the neighbourhood.

This study was inspired by microsociology and the works of Goffman [30] and Becker [31]. This framework is used throughout the design including the development of an observational guide, interview guides and coding and the analysis of data.

Our study took place in three cities, Copenhagen, Odense and Aarhus, covering the five existing DCRs in Denmark, each comprising distinct types of DCR clients. The qualitative data was collected from March 2014 to September 2014. The first part consisted of participant observation. This took place from March 2014 to June 2014 and was conducted in all five Danish DCRs. It comprised of 10 days at each site, 4–6 h per day, and in total, approximately 250 h of observational research. The time schedule for days of the observation and interviews were coordinated with the staff members and the researchers negotiated their position with the staff and where to be situated in the rooms [32]. The researchers adopted the position best suited depending on the site possibilities. The participatory role of the researcher varied from active participant with some involvement, handing out paraphernalia to the clients, to being the observer as participant with limited involvement with clients [30, 31, 33]. An observation guide was used, and focus was both on staff and clients as well as the surroundings. Topics in the observation guide included health and harm reduction-related interactions and relationship building. Participant observation took place in the DCRs and in the waiting areas of DCRs.

After the observations, the researchers wrote the field notes and divided them into descriptive, analytic and reflective categories [34]. The researchers observed interactions during the opening hours of each DCR, including weekends, holidays and evenings, in order to monitor variation in activity. This closeness enabled the researchers to become acquainted with the DCR clients and members of staff. The existing relations between staff and clients mediated contact between researchers and clients. Towards the end of the observation period, certain patterns of interactions and daily activities were identified. Clients and staff were becoming familiar with the researchers and recruitment for interviews started. The staff were helpful in answering client’s questions about the researchers’ presence and purpose, and several clients were offering to be interviewed without being asked. Through the initial analysis of the field notes, areas for in-depth interviews were identified and two semi-structured interview guides were developed, one guide for staff members with focus on work experience, job challenges, primary function in DCR, building relationship, bridge building, meaning of DCRs for clients, overdose treatment and the possibilities for harm reduction initiatives. The interview guide for clients focused on how DCR clients experienced the DCRs, and how they experienced the help they receive, their experiences of using the DCRs and how it affected their health and well-being, and how they experienced transferral to other sectors and demographics.

The second part of the data collection consisted of qualitative interviews. It comprised of semi-structured in-depth interviews with 42 DCR clients and with 25 staff members, conducted from April to September 2014. All members of the staff came from the DCR in Aarhus and the mobile DCR in Copenhagen, and two thirds of the staff in Odense were interviewed. At the two remaining DCRs in Copenhagen, we interviewed one third of the staff (*n =* 10). Regarding the recruitment of staff, we purposefully selected the staff with a variety of work experiences, ages and educational backgrounds. Out of the approximately 500 daily DCR clients, we interviewed 42. The majority had Danish nationality (see Table 2). To gain heterogeneity, we recruited DCR clients of varying ages, genders, nationalities and life and drug experiences and spreading across the facilities. Only DCR clients who understood Danish or English were included in the study. We interviewed clients in close proximity to the DCRs, in nearby offices or outside in the open air. The interviews lasted from 15 to 60 min and were digitally recorded. We recruited DCR clients who were willing to participate (convenience sampling) [35]. Some clients declined to participate, offering explanations such as being too busy or too sick.

**Table 2:** Characteristics of the sample

	Qualitative interview participants	Aarhus	Odense	Mobile unit: Fixelancen	Men’s Home: Skyen, Halmtorvet	
Total number of members of staff	25	4	5	5	11	
Median age (range)	38 (24–64)					
Gender						
Female, *n*	17	4	3	2	7	
Male, *n*	8		2	3	4	
Educational background						
Nurses	11	1^a^	0	5	4	2
Nursing aides	4	2	2		0	
Social workers	4	1	2		1	
Social educators	5	1	1		1	2
Others	1				1	
Total number of DCR clients	42	5	10	5	22	
Median age (range)	41 (22–60)					
Gender						
Female, *n*	8	0	3	1	1	3
Male, *n*	34	5	7	4	7	11
Nationality						
Danish	35					
Scandinavian	2					
Other Europeans	2					
North African	3					

^a^This nurse is both nurse and social educator

The researcher team consisted of three senior lecturers with nursing backgrounds. Two of the researchers had no prior experience in the drug-using field. One researcher has a PhD in the field of drug use and has formulated the research questions and the study design in collaboration with an associate professor of the faculty.

### Data analysis

All interviews were digitally recorded and transcribed verbatim. Transcripts were checked for accuracy, and any personally identifiable information was removed. The interviews and field notes were thoroughly read and reread and initially coded by all members of the research group; codes and themes were discussed in the research group and the transcribed interviews and field notes were entered into the NVivo program [36], a software package designed to organize and code qualitative data. As the process of analysis proceeded, new codes that were more refined and specific were added. During the process of analysis, we identified the meaning units that eventually became codes [37]. The research was inspired by a microsociological perspective with focus on interactions between staff and DCR clients. Concepts of stigmatization and deviance were important perspectives during analysis. The material was coded and the following three main themes were developed: (1) social acceptance in a secure environment; (2) survival, health and well-being of DCR clients; and (3) building bridges between DCRs and other sectors. In the analysis presented in this paper, we have integrated and combined all qualitative material, and excerpts from interviews and field notes will serve to exemplify and support the themes.

## Results

**Table:** 

Themes	
1. Social acceptance in a secure environment 1.1 Non-judgemental approach in the DCRs 1.2 How staff forge relationships and trust in a hectic environment 1.3 Challenges in the DCRs—seeking normality in a hectic environment	
2. Survival, health and well-being of DCR clients 2.1 Prevention of overdoses 2.2 Physical health: hygiene, wound care and injection technique 2.3 Mental health: providing care and protection	
3. Building bridges between DCRs and other sectors 3.1 Drug treatment and the social sector 3.2 Referrals to the healthcare sector	

### Theme 1: social acceptance in a secure environment

In this section, we explore the manner by which members of the staff try to cultivate an environment of social acceptance inside the DCRs and how staff members forge relationships with DCR clients. We then examine the challenges experienced by both staff and clients in the interpretation and upholding of house rules.

#### Non-judgemental approach in the DCRs

DCRs constitute low-threshold harm reduction facilities. Their mission is not to rehabilitate their clients but rather to reduce potential harm, especially that which is caused by incorrect drug use and injection techniques. To fulfil this end, DCRs must ensure that DCR clients utilize the facilities appropriately and follow basic rules and guidelines. The DCR clients interviewed at all five participating DCRs unanimously reported that they felt safe and were treated respectfully in these facilities. For example, one DCR client stated, ‘it is a really nice place where I feel welcome’. The majority of DCR clients with whom we spoke expressed satisfaction with the DCRs. They stated that the DCRs provide a safe environment for injecting and smoking. DCR clients experienced the members of staff to be welcoming, and the clients felt encouraged to engage in conversation with staff about their life circumstances and their general well-being.

It feels like a ‘safe haven’ to come here … because I know that they have an agreement with the police not to hassle us. Because normally the police chase after us, so we aren’t safe anywhere. We didn’t choose to live like this; we are forced to do things, so it is nice to have a place that serves as a safe haven, a place where nice people welcome me and where they know me, and they know what is going on (DCR client, age 44).

The staff in DCRs tried to enable clients to maintain a degree of human dignity as well as enjoy a measure of safety. There are ‘safe zones’ around the DCRs, where local police do not confiscate the illicitly obtained drugs [28, 38]. As described above by the DCR client in the interview, the DCRs function as ‘safe havens’, provide clients with safety from theft from other clients and protect them from being pursued or hassled by the police.

DCRs endeavoured to safeguard the DCR clients’ sense of dignity and self-worth insofar as clients experienced acceptance and recognition by staff members. One client said: ‘They (staff) talk to us at eyelevel. You do not have to be embarrassed that you are a drug user here’.

When interviewed about the importance of DCRs, the members of staff agreed with this description. They explained how the introduction of these facilities has improved the well-being of the clients, creating a sense of dignity and preventing unnecessary deaths due to ODs. As one of the nurses said,

But there’s no doubt that for the drug users this is a really, really good step in the right direction. Before they used to shoot up outside in the cold, in staircases, or in playgrounds using water from puddles. They shared syringes and they lived miserable lives. For many years they have been crying out: ‘Give us a place now. Maybe I cannot help using drugs but give me a decent life and some dignity’…. It has been horrible for them. So I think that it means a lot to get off the streets, and to not be looked down on by other people. I believe that safety is very important. Many of them have overdosed or have seen their friends overdose. Many have lost friends in the drug scene. So in that sense I think it means the world to them (Staff member, RN, age 32)

As the nurse stated, DCR clients have felt exposed when injecting in public which might have led to feelings of being outsiders and stigmatized by society [30, 31]. Several of the clients recalled how life used to be before the opening of DCRs, with stressful situations in basement stairwells or in public spaces, where they risked disturbing others and were often chased away. However, when asked where they consume drugs when DCRs are closed, DCR clients replied that they still make use of their homes or public spaces such as toilets, parks and hotels.

#### How staff forge relationships and trust in a hectic environment

According to members of the staff, establishing relationships and building trust with DCR clients is a responsibility that is central to the effectual operation of DCRs. The staff’s priority is thus to gain the trust of the clients; once this is achieved, the staff focus on encouraging clients to seek support and assistance beyond the DCRs. From a field note:

The staff told me (the researcher) afterwards about Max; they noted that he has exhibited very violent behaviour. It has taken them a long time to be able to talk with him in the way they are able to do now. He has spoken with the staff about his upbringing; he was at multiple foster homes, and was repeatedly exposed to violence and abuse (Field note 21.03.14).

Establishing trust with DCR clients can be challenging due to the typically brief periods of time that staff are able to spend with the clients. There was a continuous flow of clients entering and exiting the DCRs during the facilities’ busiest hours, which only allowed for very limited interactions with each individual. These time constraints posed a challenge because, as one nurse noted, establishing trust takes time:

First of all, it took more than a month before I was accepted, and after coming back from vacation they (DCR clients) realized that I was more than a temporary staff member, and they began to ask for me by my name. So you start to lend a hand or put your arm around someone’s shoulder and ask ‘How are you doing today?’ Or they watch you care for one of their friends, and they remember this. And gradually, the more I have helped, the easier it becomes to set limits, and they do as I tell them. And they realize that I am not always annoyed. So, really, you have to give, give and then you will be able to take a little bit (Staff member, RN, age 42)

Gaining acceptance and trust of DCR clients is thus a time-consuming endeavour. As remarked by the nurse in the quote above, members of the staff must be willing to give a great deal in order to establish relationships and to be able to make demands with clients, who as a consequence of a life with stigmatization and marginalization may feel inherently distrustful and guarded. To give and take in this quote emphasises the importance of reciprocity between the clients and the nurse, and this is necessary for developing relationships. To build relationships and engage in conversations seems to have better conditions in the smaller facilities as they are not as busy as the bigger DCRs.

#### Challenges in DCRs—seeking normality in a hectic environment

At times, both members of the staff and DCR clients experienced challenges. On the one hand, a number of clients criticized DCRs for being too noisy and conflict-ridden. In some cases, these factors deterred clients from utilizing the DCR, leading them to opt either for the mobile DCR or for the street. On the other hand, it is a balance to keep the DCRs as low-threshold facilities. Certain clients emphasised that the house rules ought to be respected and, if they are not, that the staff should impose sanctions on those failing to observe the rules. One female client said:

They often talk too loud and I think they could have a rule such as - if you shout, then you have to leave. Because many are here to consume coke and they want it to be quiet. And if they are shouting at one another then it kills the buzz. So I think if you can’t shut up, then you should leave (DCR client, female participant, age 48)

It was clearly challenging for members of staff to strike a balance between maintaining a safe and tranquil environment within the DCR, while at the same time reaching out to and restraining DCR clients who exhibited inflammatory and antagonistic behaviour. As one of the nurses put it:

I used to work in a psychiatric ward where there were rules, cleanliness and consistency. So, in my opinion, the rules here are a bit vague as to when individuals should be put under a temporary ban – some staff members are very tolerant while others are stricter. So this leaves room for disagreement, and it is confusing for the clients as well as for me as a staff member (Staff member, RN, age 42)

The nurse pointed out that the rules were difficult to administer as they were not clear and standardized thereby opening for potential conflicts. She requested that the rules were more standardized, which in her opinion would benefit the staff and clients alike. Several clients were in agreement. In one interview, a male client put it as follows:

We could use representatives - one for the smokers and one for the junkies. He could sort of say: “Hey, listen to the staff”, acting as a kind of authority figure (DCR client, male, age 60)

As the DCR client pointed out, a spokesperson could mediate between clients and staff and support the staff by encouraging that everyone respects the rules in order to make the room calm and safe for injecting or smoking. Some clients were very helpful and helped maintaining peace and tidying up after themselves and other clients.

### Theme 2: survival, health and well-being of drug users

Preventing and treating overdoses is one of the most important aims of the DCRs. In this section, we start by exploring the informational and interventional strategies that members of staff employ to prevent ODs. We then focus on the general impact the DCRs have on promoting physical health, e.g. hygiene, wound care, injection techniques and mental health, e.g. providing care and protection.

#### Prevention of overdoses

The foremost goal of DCRs is to prevent ODs and deaths caused by ODs among DCR clients. During the course of our study, we observed that the DCR staff employ both informational and interventional strategies to achieve this goal.

#### Dissemination of information regarding the strength of drugs in circulation

The informational strategy of DCRs consists of staff members gathering current information from DCR clients about the strength of the drugs in circulation, and subsequently, if they are aware of potent drugs circulating on the market, they warn clients. We observed that the staff were attentive as to which drugs were in circulation at any given time, as well as to how potent they were. If the staff observed or were informed by clients that drugs on the street were strong, they cautioned other clients and suggested reducing doses by half in order to prevent overdoses. One client who was visiting the DCR for the first time was given such a warning. The nursing aide informed him that the heroin might be very potent, and she advised him to start with a half dose. He later conveyed to the aide that he had become very ‘hit’, and he stayed at the DCR for some time until he felt less intoxicated. Another client had a suggestion to ameliorate the DCRs:

I know from Holland that they have quality places where you can have your drugs tested. It would be nice to have such a place here where the quality and the strength could be checked (DCR client, male, age 44).

#### Intervention

The observational studies showed that the interventional strategy focuses on the consistent observation of clients in the DCR, so as to be in a position to detect whether any individual appears to be at risk of an OD. In cases of high opioid consumption, the staff upgraded their monitoring of the individual, talking to him or her, inquiring as to how the client was feeling, and advising him or her not to consume further doses for the time being.

As one nurse explained:

We wait with the naloxone administration until they become unconscious and don’t breathe because we know that we can help them in that case. That’s why we let them sit and chill. Maybe we stimulate them, talk to them, shake them, pain stimulate so they wake up and remember to breathe. (Staff member, RN, age 52)

Another nurse elaborated on the interventions:

If they saturate under 90 %, we give them a little oxygen under the nose and make sure that they breathe. Maybe we support their head and chin to secure airways. And we let them chill as long as their vital signs are OK. It is not so dramatic. In that way we do not ruin their fix and the rest of the day (with Naloxone) (Staff member, RN, age 32).

In severe cases in which clients experienced respiratory problems or became unconscious, the antidote naloxone was administered as instructions prescribe neutralize the effects of the opioid taken [39]. Naloxone was unpopular among some of the clients as it dulls the high, and clients may experience opioid withdrawal symptoms when they come down from the high. The DCR staff did not always readily administer naloxone as they were aware that the half-life of naloxone is shorter than that of opioids, which means that the effect of the opioid may return after the client has left the DCR, and the client could potentially be at risk of an overdose once out of reach of the facility.

Cocaine overdoses seemed to be more complex to handle as clients, who use cocaine, exhibited different behavioural symptoms. Some had stereotypical repetitive behaviours where they were looking for things or had psychotic symptoms, for example, experiencing sensations. The behaviours lasted from minutes to hours where the staff observed closely, carefully monitoring the clients’ blood pressure and pulse, eventually treating them with acetylsalicylic acid and nitro-glycerine spray when deemed appropriate [39].

#### Cultivating a sense of security and preventing overdoses

The presence of trained personnel, ready to intervene should something go wrong, provides DCR clients with a sense of safety and the opportunity to feel at ease. Many clients have experienced overdose situations prior to the opening of the DCRs, so they are aware of the importance of consuming drugs in safe environments. One DCR client emphasised why he prefers to inject in the DCR:

If I get something too strong and risk an overdose, it is a safe place. They will always be there to help. And the equipment is sterile, so I don’t risk contracting staphylococci or Hepatitis C (DCR client, male, age 44)

Being in secure surroundings is important for the DCR clients both in relation to survival of an overdose and the prevention of infections. Another client witnessed an episode in the DCR, in which an intoxicated client entered the facility. The client commented on the episode:

It was very unpleasant today… there were two near overdoses, and then he talked about taking more drugs. Then I opened my mouth and said that he shouldn’t have more drugs because then they will kill you. You are not going to die in there (DCR client, female, age 48).

Many of the DCR clients had experienced overdose situations and had lost friends before DCRs opened, so they are aware that it is imperative to consume drugs in a protected environment.

#### Physical health: hygiene, wound care and injection techniques

Staff members also focused on monitoring hygiene and risk practices related to the transmission of infections among DCR clients, as this could prevent complications and reduce the risk of severe disease. Whenever possible, the staff encouraged good hygiene and hand washing. They made these suggestions gently, in such a way so as not to dismay or unnerve users. Staff members, particularly healthcare professionals, monitored DCR clients in order to detect any signs of infection. They advised the clients to seek medical assistance, and in severe cases, they either referred the clients to nearby health clinics or, if necessary, to specialized treatment and surgery at hospitals. One nurse said:

For some it might be difficult to understand but in my opinion there’s so much nursing here. For example, today I rinsed the eye of someone who had some alcohol thrown into the eye. It can be mental nursing - conversations. Staying and talking, listening and accepting, and setting boundaries. A little wound care, injecting techniques. People sometimes cut themselves or somebody cut them. So there is so much nursing if you know what to look for (Staff member, RN, age 42)

Although wound care is normally not offered in DCRs, the nurse indicated that the help offered involves many different aspects of nursing. Our observations revealed that nurses were able to intervene in a variety of ways. They invited the DCR clients to visit the local clinic to have their wounds treated. On one occasion, the nurse failed to persuade the client to visit the local clinic, so she contrived a makeshift bandage on the spot, right on the street in public view outside the DCR. Some clients sought medical assistance from DCR staff because they trusted them more than other health professionals. In one instance, a client with a deep ulcer on her hand, which came as a result of injecting cocaine incorrectly, was asked whether she had received any help from the staff. Her response was as follows:

The doctor is going to have a look at my fifth finger. The staff told me that the doctor at the clinic nearby could look at it. They wanted me to go to the hospital, but I refused because then the city administration and my GP will know about it, and I don’t want them to know. And they (the staff) helped me to get antibiotics, so I had two kinds of AB, and the wound has now become half the size. (DCR client, female, age 48)

Our observations in the DCRs indicated that staff members were attentive to the hygiene of clients. The members of staff encouraged DCR clients to use new needles at each skin penetration, and fortunately, this resource was unlimited. However, some clients had developed unsafe and unhygienic habits during their active years of drug use, and they often found it difficult to locate veins. Safe injection techniques are important skills in a harm reduction framework. For this reason, all DCRs are equipped with a vein scanner to locate veins for safer injecting practice in order to find suitable intravenous access. The members of staff suggested other drug intake methods if the DCR client could not find veins, such as taking the drug orally or intramuscularly. Still, most clients prefer to inject intravenously, and their long-established habits are frequently difficult to change.

#### Mental health: providing care and protection

It is widely acknowledged that people who use drugs experience a variety of mental health issues in addition to their addiction, yet not all have been diagnosed, and many do not receive relevant treatment. The members of staff in DCRs often functioned as aides and witnessed DCR clients who seemed to have mental health conditions. The staff supported and cared for clients whether the clients’ hallucinations and paranoia were due to drugs or the drugs were taken to mitigate the symptoms of their conditions. The following excerpt illustrates how caretaking was carried out in one DCR:

A tiny woman is cuddled up on the floor in the corner panting and looking panicky around in the DCR. She wants the window closed although it is around 30 °C in the room, apparently afraid that something threatening might come through the window. Both nurses are near her. One of the nurses helps her up from the floor. Her forehead is beaded with sweat, and one of the nurses asks her: “Can I dry your face?” She asks: “Can I do it myself?” The nurse hands a paper tissue moistened with cold water. The woman crawls down on the floor again; she obviously feels safer there (Field note 23.05.14).

In this case, two nurses cared for the woman by reassuring her that she was safe and secure, while offering her practical help, assessing and safeguarding her from other clients in the room. They were caring towards her and offering her assistance within the scope of the DCR. In another instance, the members of staff referred a psychotic client to the psychiatric emergency room (PER). In this case, a female regular client became increasingly aggressive, shouting at and admonishing those around her; she provoked conflict, expressed frustration and felt sick, and eventually the staff were not able to take responsibility for her security and health within the DCR. The members of staff organized for her to visit the PER once she had injected her drug, since she had psychiatric problems in addition to her addiction. The staff’s previous experiences with the client enabled them to assess her condition and determine that a referral to the PER would best protect the woman; she agreed to go to the PER, but the psychosis subsided and, within a few hours, she was discharged from the PER and returned to the DCR. This is one case of many, during the course of this study, in which the staff tried to provide assistance for mental health problems.

### Theme 3: building bridges between DCRs and other sectors

DCRs are expected to build bridges to other health institutions [28]. DCR members of staff are in daily contact with DCR clients and are thereby in a position to both motivate and assist clients, e.g. seek treatment for somatic or psychiatric conditions or to take steps towards commencing drug treatment. Despite the fact that clients visit DCRs anonymously, the staff often managed to successfully establish relationships with clients and to acquire information regarding, e.g. how to motivate particular clients to seek treatment, the clients’ housing situations and their social conditions. In our study, we observed and spoke with DCR clients and staff about forging links to drug treatment centres, the social sector and the health sector.

#### Drug treatment and the social sector

DCRs are low-threshold facilities, and providing referrals to drug treatment is not the primary mission of the staff. Many of DCR clients in our sample were in opioid substitution therapy, either with methadone, heroin or subutex, and the drugs taken in the DCRs might be considered extraneous abuse. However, we observed that staff attempted to assist those clients who were motivated to undergo drug treatment by contacting treatment centres or by connecting these clients with social workers on outreach who could initiate treatment on the spot if the individual was so inclined. The members of staff reported that social workers were able to provide contact with acute treatment facilities. The main ways in which members of staff encourage clients to seek treatment was through regular conversations, continuous encouragement and nudges.

We were talking about Tai (a female DCR client). I hope that we (the DCR staff) have helped to push her in the right direction, although she wasn’t hospitalized through us … But I hope that we have planted some small seeds here. Sometimes we are the ones that believe in them, and say: “Well, you can do it, you can easily do it. If you don’t succeed this time, then next time.”- and we are here if they relapse (Staff member, nursing aide, age 28).

This nursing aide described the approach through which members of staff try to nudge clients whenever the opportunity arises, in order to motivate and encourage them to commence treatment programmes. She used the analogy of planting seeds when discussing the motivational conversations that took place. It was clearly of concern to the members of staff that they instil in DCR clients a sense of the importance of treating addiction and also that, if the clients relapsed, this was not regarded as a failure, and the members of staff would be there to pick up where they left off and would continue helping.

When asked whether a dialogue may push a client towards treatment, one nurse answered:

Yes, certainly. One thing is that when they are sitting around talking, they share many different personal problems. But otherwise, as nurses we try to guide the conversation in the right direction, or we focus on the possibilities instead of the limitations, and we look at their strengths rather than their weaknesses. And if they have an idea that seems to be very, very good for them, then we try to stick to it and find out whether we can help them further. For example, if they want to enter into treatment or care or whatever they want, we try in every possible way to refer them, or we contact the social workers or whoever can help them in the given situation (Staff member, RN, age 32)

This excerpt shows that the tasks of the staff in DCRs are multi-faceted: pulling the right strings, pushing in the right direction and expressing a positive attitude towards the clients. Some DCR clients had complex problems to deal with, and as some DCRs had social workers on staff, they were able to help clients with socioeconomic problems such as bills, pension and housing. In some cases, social workers did outreach in the DCRs or members of staff referred clients to social workers at nearby health clinics or to public defenders.

#### Referrals to the healthcare sector

People who use drugs often contract skin infections due to unhygienic and incorrect injection techniques. To manage wounds, infections and so on, the staff referred DCR clients to health clinics. However, some clients neglected their own health and postponed treatment, where other clients showed more motivation by attending the DCRs in order to protect their health.

One nurse, for instance, pointed out that sometimes DCR clients were not interested in or too busy to seek treatment. She noted that it would be beneficial if staff were authorized to treat wounds on the spot in the DCRs, because at times, they noticed clients ignoring large wounds and other chronic conditions. Staff also referred clients to their general practitioners, but it is uncertain to what extent DCR clients followed up on these referrals. One of the nurses described the dilemma: ‘I have many good relationships but it is not possible to find out if the bridge building was successful. Because when the client leaves I can’t pursue him (Staff member, social educator, age 42).

Because making appointments is not necessary in order to receive medical attention at health clinics, they are easier for clients to frequent, and they function as outreach centres for the most vulnerable. Most referrals made in DCRs were to health clinics in proximity of the facility, but sometimes referrals to emergency rooms were necessary.

## Discussion

Our findings from Denmark supplement previous studies [21] confirming that DCRs provide safe, stress-free environments for DCR clients in which they are sheltered both from the police as well as from other clients [40]. Our study reveals that the humanizing approach of DCR staff, combined with the provision of facilities and tools for drug consumption, appear to promote a sentiment of social acceptance among DCR clients. Our findings are similar to those of Rance and Fraser [23] in what they term ‘accidental intimacy’. They also find that a non-judgemental and humane approach employed by the DCR staff empowers the DCR clients towards feeling more ‘like citizens rather than scummy junkies’ [23]. This seems to constitute the most important feature of DCRs and is that which ultimately paves the way for both the successful prevention of ODs, as well as for steering clients towards utilizing both social and health services, including drug rehabilitation facilities. This investigation also corroborates results of other studies demonstrating that acceptance of drug clients is an essential step towards de-marginalization and de-stigmatization [23, 24, 41].

Another result reveals an institutional barrier that may be intrinsic to the very concept of providing low-threshold services to a group that is both disadvantaged and heterogeneous. On the one hand, the containing of all DCR clients represents the essence of low-threshold facilities, which in this context means containing clients who are subjected to chaotic circumstances and whose ability to adjust becomes impaired. Yet on the other hand, DCR clients constitute a heterogeneous group such that some clients are more stable than others. As a consequence, some DCR clients find it difficult to be in the DCRs where the environment can sometimes be calamitous, which may deter them from utilizing the facility. More rules, or following existing rules more rigorously, may not solve this problem either, since this might scare off the more vulnerable DCR clients and would thus conflict with the stated intention of low threshold.

Also, our study indicates that the Danish DCRs employ a twofold strategy comprised of an informational and an interventional-preventative component. The first element of the preventative strategy consists of being apprised as to which drugs are available, how potent they are and in disseminating this information to the DCR clients, utilizing the facilities. In a number of instances, we observed that this strategy was effective in preventing potentially perilous or fatal situations from transpiring. However, a more precise way to test the drugs would be on-site drug testing [42] which one of the DCR clients in the sample mentioned and advocated for. The approach was successful due to the trust forged between the DCR staff and clients, who willingly heeded staff’s advice on being cautious as to their intake. The second element of the preventative strategy consists of actual intervention by providing clients with naloxone. Our study revealed that this latter part of the preventative strategy posed DCR staff with a complex dilemma due to the two considerations at play when staff must determine whether to administer naloxone. Administering this pharmaceutical may save the life of a client who would have otherwise overdosed. However, doing so will inevitably lessen the DCR clients’ ‘hard-earned’ high. In effect, we observed that while norms as to the circumstances under which naloxone should be administered varied among both the personnel and the DCRs, in general, the member of staff went to great lengths to avoid administering the pharmaceutical because obstructing the clients ‘rush’ was an extremely delicate situation and also detrimental to the relationship between the staff and clients. A recent Danish ministerial evaluation of DCRs indicates that the implementation of DCRs in Denmark has had an effect on reducing the number of deaths by overdose in the cities that have implemented the DCR programme [43].

Our study confirms that the work of the healthcare professionals in DCRs effectively aides DCR clients in employing safer and more hygienic injection techniques, which in turn reduces the number of incorrect injections that result in infections [6]. Still, it is unfortunate that the nurses are not allowed to provide basic medical care directly inside Danish DCRs, as this is practiced in other DCRs and has been shown to be very efficient [6, 24].

DCRs function as bridges to other health and social services, including drug treatment. The DCRs refer clients to a variety of treatment and healthcare facilities whenever possible. However, as it is the responsibility of clients to follow up on these referrals, it is not possible, in this study, to demonstrate what motivates the clients to seek more treatment or help. The registration of DCR clients is through aliases, making it impossible to track whether the bridge building has a meaningful effect.

In sum, our study emphasises the potential of fostering a sense of social acceptance of DCR clients as a platform for both improving their health and well-being, as well as for encouraging these clients to take steps towards risk-reducing drug intake behaviour.

## Conclusions

Our study from Denmark confirms the findings of similar international studies. The study reveals that there is consistency between DCR clients and members of staff with respect to their appraisal of the importance of Danish DCRs. Both clients and staff emphasised how DCRs provide a safe haven from the often chaotic environments which they tend to face outside on the street. The DCRs shelter clients from the police as well as from other clients and provide these individuals with a setting in which they may take drugs with clean tools under relatively calm conditions. DCR clients stressed that the approach of DCR staff was crucial to creating a welcoming and humanizing atmosphere. The staff members interviewed were also very cognizant of the significance of fostering this friendly climate. Still, some clients and staff members noted that they would appreciate more sanctions for clients who shout or display other types of subversive behaviour. Despite the brief and hectic meetings, and the DCRs being noisy and conflict-ridden at times, the circumstances of DCR clients have according to both the clients and staff been improved as compared to their lives prior to the opening of DCRs.

### Limitations of the study

While we believe it to be strength of the study that clients from each of the five Danish DCRs have participated, we were unable to interview all types of clients due to language barriers as several DCR clients did not speak Danish or English. Moreover, as previously noted, certain clients declined to participate due either to illness or a variety of other reasons.

### Further research

Based on our study, we believe that future research ought to address the following two questions. Firstly, the transition of clients from DCRs to primary and secondary care does not appear to have received sufficient examination in the Danish context. Previous studies [44] support the notion that stereotyping people who use drugs is pervasive in primary and secondary care. Therefore, it would be beneficial to investigate clients’ transition from DCRs into primary and secondary care, in order to discern which impediments and facilitators may be in play. Finally, among the most vulnerable clients in DCRs are those who suffer from mental problems in addition to their use of drugs. This raises the question of whether the DCRs have potential to develop custom-made measures to better assist this subgroup of clients.

## Abbreviations

DCR, drug consumption room; OD, overdoses; OST, opioid substitution treatment; PER, the psychiatric emergency room

## Footnotes

1 The Men’s Home has a 100-year long history of charity work for homeless people. The institution has many different offers; the housing section is solely for men whereas both genders can use the health clinic, the open counselling and DCRs.

2 Danchurchsocial is funded on Christian philosophy with charity and a human but not proselytizing orientation towards socially marginalized groups in society.
